# The Role of Macrophages in Endometrial Cyclical Changes and Female Reproductive System Diseases: A Review

**DOI:** 10.3390/cimb48080832

**Published:** 2026-08-17

**Authors:** Shuyuan Zhang, Luyang Zha, Chenyuan Liu, Aijia Wang, Yaxin Guo, Kun Qian

**Affiliations:** 1 Reproductive Medicine Center, Tongji Hospital, Tongji Medical College, Huazhong University of Science and Technology, 1095 Jiefang Avenue, Wuhan 430030, China; 2 Department of Ophthalmology, Tongji Hospital, Tongji Medical College, Huazhong University of Science and Technology, 1095 Jiefang Avenue, Wuhan 430030, China

**Keywords:** macrophages, endometrial physiology, menstrual cycle, pregnancy, reproductive immunology

## Abstract

**Background**: This review aims to systematically summarize the lineage origins, subtype classification, and functional dynamics of endometrial macrophages, clarify their mechanisms of action in normal reproductive physiology (menstrual cycle, pregnancy) and common reproductive diseases, integrate cognitive breakthroughs brought by cutting-edge research technologies, and provide theoretical support for basic research and clinical translation in reproductive medicine. **Methods**: Recent basic and clinical research studies related to endometrial macrophages were retrieved, with a focus on incorporating findings from technologies such as single-cell sequencing and multi-omics. The reviewed content covers core aspects including macrophage origins (embryonic-derived, bone marrow-derived), subtype classification (M1/M2 and novel metabolism-related subtypes), cycle- and pregnancy-specific functions, and disease-associated mechanisms. A comprehensive analysis of the regulatory networks of endometrial macrophages under physiological and pathological conditions was conducted. **Results**: Endometrial macrophages, by virtue of their phenotypic plasticity and functional heterogeneity, play a central role in cyclical endometrial remodeling, pregnancy establishment, and the regulation of reproductive immune homeostasis. Dysregulation of their function is closely associated with various reproductive disorders such as recurrent spontaneous abortion and endometriosis. In-depth exploration of their biological characteristics and regulatory mechanisms holds great significance for filling knowledge gaps in the field of reproductive immunology and advancing precise prevention and treatment of related diseases. **Conclusions**: Endometrial macrophages are core regulators of the reproductive immune microenvironment, and their spatiotemporal dynamic functions are closely linked to reproductive health. The revelation of novel classification systems and regulatory mechanisms provides new perspectives for in-depth understanding of reproductive physiological and pathological processes, as well as important targets for immune-targeted therapy of reproductive-related diseases. This holds great clinical translational significance for promoting the precision development of reproductive medicine.

## 1. Introduction

Macrophages are highly plastic innate immune cells derived from bone marrow monocytes and are widely distributed in body tissues [1]. As a core immune system component, they clear pathogens and cellular debris via phagocytosis and also maintain tissue homeostasis, regulate inflammation, and promote tissue repair [2]. Guided by microenvironmental cues, macrophages polarize into pro-inflammatory M1 or anti-inflammatory M2 phenotypes, showing significant functional heterogeneity [3]. Even under sterile conditions, tissue injury induces rapid macrophage recruitment—reversible upon repair completion—indicating functions beyond simple phagocytosis [4]. Recent studies highlight that tissue-resident macrophages are primary immune responders in injured microenvironments. By secreting chemokines, cytokines, growth factors and other effector molecules, they initiate local inflammation and precisely regulate transendothelial migration of myeloid cells (e.g., neutrophils) [5], acting as key modulators of microenvironmental remodeling.

The endometrium is a dynamic, hormone-responsive tissue that undergoes nearly 400 cycles of proliferation, secretion, and shedding during a woman’s reproductive life [6]. It consists of a regenerative basal layer and a cyclically changing functional layer, tightly regulated by estradiol and progesterone: estradiol drives endometrial growth in the proliferative phase, while progesterone induces decidualization (stromal cell differentiation) for embryo implantation in the secretory phase [7]. In the absence of pregnancy, progesterone withdrawal triggers menstruation, involving ischemia, inflammation, and tissue shedding. Meanwhile, macrophages and other mediators promote repair and regeneration to ensure efficient endometrial reconstruction [8]. This remarkable process relies on the precise coordination of a complex hormone–immune–vascular network.

**Figure 1:**
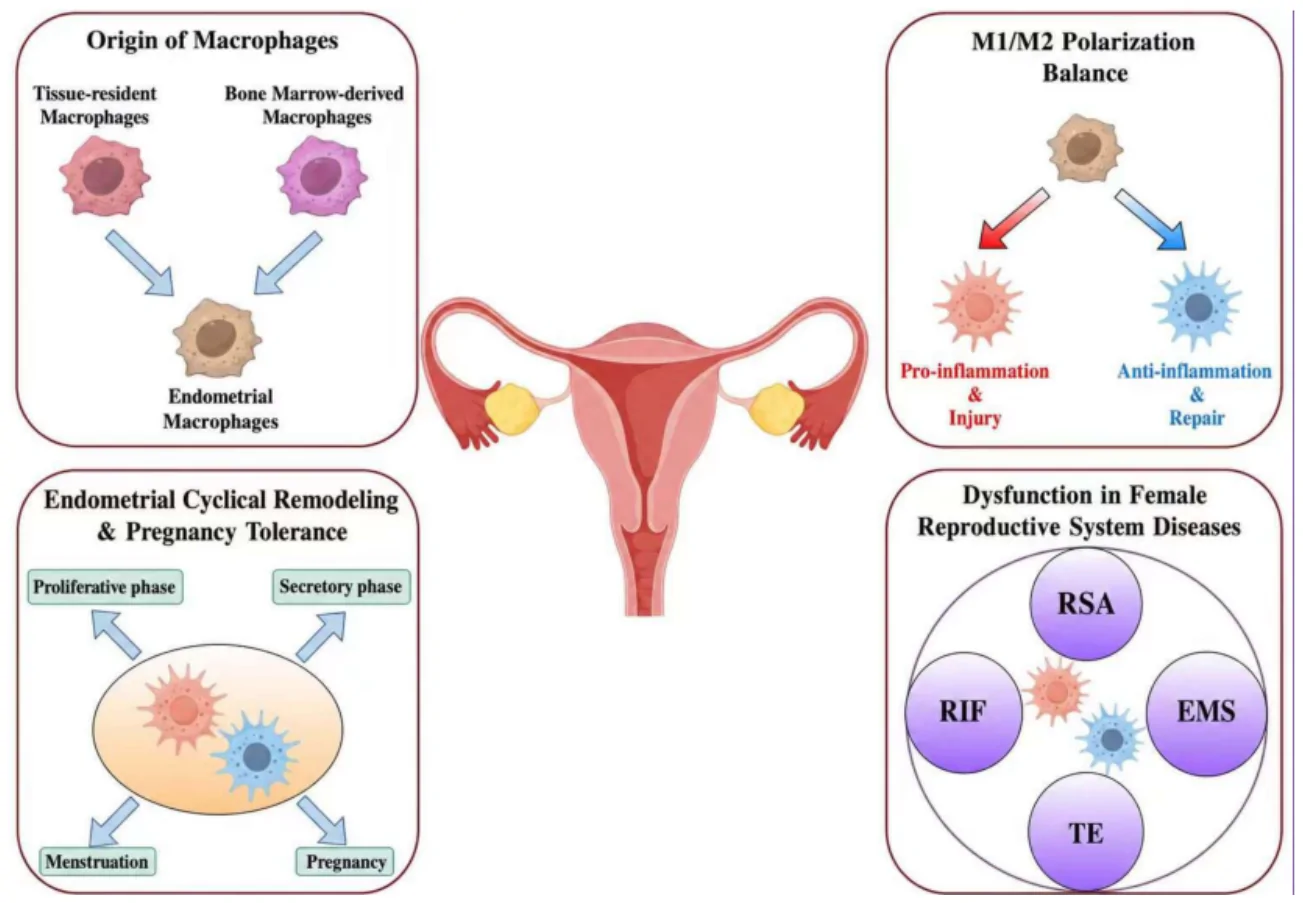
This review systematically summarizes the origin, polarized subtypes and functional heterogeneity of endometrial macrophages, highlighting their roles in endometrial cyclic remodeling, maternal–fetal immune tolerance during pregnancy, and associations with female reproductive system diseases. Abbreviations: RSA = recurrent spontaneous abortion; RIF = recurrent implantation failure; EMS = endometriosis; TE = thin endometrium.

Although endometrial macrophages play pivotal roles in menstrual repair, immune regulation, and decidualization, their precise phenotypic characteristics and functional mechanisms have not yet been fully elucidated. This review focuses on elucidating the functions of macrophages in both normal endometrial physiology and pathologies of the female reproductive system, with particular emphasis on their dual roles in tissue homeostasis and disease pathogenesis (Figure 1).

## 2. Endometrial Macrophages

Macrophages are innate immune cells widely distributed throughout bodily tissues, capable of clearing pathogens and foreign bodies through chemotactic migration and phagocytosis [9]. As highly plastic immune cells, macrophages also participate in critical processes such as immune regulation and tissue repair, playing a central role in maintaining organismal homeostasis and disease modulation [10].

In the endometrium, macrophages are the second most abundant immune population after natural killer (NK) cells. With their phenotypic plasticity, they play a pivotal role in maintaining tissue homeostasis, mediating inflammatory defense, and regulating reproductive processes—including cyclic remodeling and immune tolerance during pregnancy [11]. Dysregulation of their function is closely linked to various reproductive disorders. Thus, deeper elucidation of the biological characteristics of endometrial macrophages has significant scientific and clinical value for understanding immune regulatory mechanisms in female reproduction.

### 2.1. Lineage Origins

#### 2.1.1. Embryonic Origin

Embryonically derived macrophages colonize the endometrium during early development and are postnatally maintained mainly via self-renewal, with minimal contribution from bone marrow hematopoietic stem cells—thus they are termed tissue-resident macrophages (TRMs) [12]. These cells play a pivotal role in preserving tissue structural integrity and physiological function. Based on developmental origins, endometrial TRMs are classified into two distinct lineages: one from yolk sac-derived myeloid progenitors (EMPs) that migrate to the uterus during embryogenesis and persist long-term; the other from fetal liver hematopoiesis-generated monocytes that infiltrate the endometrium via circulation and differentiate [13]. Single-cell RNA sequencing studies by Bian et al. have confirmed that the developmental origins of human embryonic macrophages are highly conserved with murine models [14]. Both TRM populations exhibit substantial self-renewal capacity and are primarily involved in maintaining tissue homeostasis and immune tolerance [15].

#### 2.1.2. Bone Marrow-Derived

Bone marrow-derived macrophages originate from bone marrow hematopoietic stem cells, progressing through common myeloid progenitors (CMPs) and granulocyte-monocyte progenitors (GMPs) to mature into monocytes that enter the peripheral circulation. Guided by chemokines (e.g., CCL2, CX3CR1) and hormonal signals (estrogen, progesterone), these monocytes migrate to the endometrium, differentiate into macrophages, and participate in inflammation, tissue repair, and hormonal regulation [16]. This population is subdivided into two subsets derived from classical (pro-inflammatory) and non-classical (patrolling) monocytes: the former contributes to acute inflammatory responses and tissue repair, while the latter is responsible for vascular surveillance and immunomodulation during chronic inflammation and viral infections [16]. These two subsets differ significantly in migratory properties and immune functions, cooperating to maintain endometrial immune surveillance and tissue homeostasis.

### 2.2. Subtypes and Functions

Based on developmental origins, macrophages are categorized into embryonically derived tissue-resident macrophages and bone marrow-derived monocyte-derived macrophages. Their composition and function are dynamically regulated by physiological/pathological states, hormonal cues, and microenvironmental signals. Under homeostatic conditions, tissue-resident macrophages maintain population stability through self-renewal and uphold tissue homeostasis. In pathological contexts, monocytes are recruited to injury sites, differentiate into classical or non-classical subsets, and participate in inflammatory and repair processes [16]. During cyclic endometrial remodeling, macrophages undergo finely tuned polarization, coordinating post-menstrual repair and maternal–fetal immune tolerance establishment. They thus act as pivotal immune regulators in maintaining this unique microenvironment and supporting key female reproductive processes [17].

#### 2.2.1. Classical Subtype Classification (The M1/M2 Paradigm)

Macrophages exhibit remarkable plasticity and functional heterogeneity, enabling them to respond to microenvironmental changes and undergo dynamic phenotypic transitions—a process termed polarization [18]. Polarization is the process by which macrophages, stimulated by microenvironmental signals, undergo phenotypic shifts via epigenetic and transcriptional reprogramming to acquire specific functional capacities [19]. Based on surface markers, cytokine profiles, and functional characteristics, macrophages are broadly classified into classically activated (M1, pro-inflammatory) and alternatively activated (M2, anti-inflammatory/repair) subtypes. The M1/M2 model provides a clear paradigm for investigating the roles of macrophages with distinct transcriptional profiles in immune homeostasis [20].

M1 macrophage polarization is driven by Th1-type immune response-related factors (e.g., IFN-γ, TNF-α), bacterial pathogen-associated molecular patterns (PAMPs) such as lipopolysaccharide (LPS), and myeloid cell regulator granulocyte-macrophage colony-stimulating factor (GM-CSF). This process mainly relies on signaling pathways including JAK/STAT1, TLR4/NF-κB, and JNK/Notch, ultimately producing a range of pro-inflammatory mediators. These mediators include cytokines (NO, TNF-α, IL-1β, IL-6, IL-12, IL-23) and chemokines (CXCL5, CXCL9, CXCL8-10, CXCL11). These cells highly express surface markers (CD80, CD86, iNOS, TLR2, TLR4, HLA-DR) and exhibit potent pro-inflammatory properties, tissue clearance capabilities, and Th1 immunity promotion [21,22]. In the endometrium, M1 macrophage numbers increase during the late secretory and early menstrual phases (driven by IFN-γ) to participate in clearing shed tissue and apoptotic cells [23]. Their polarization is also favored during the implantation window and parturition due to enhanced inflammatory processes [24,25].

In contrast, M2 macrophage polarization is induced by stimuli including Th2-type immune response-related cytokines (e.g., IL-4, IL-13), IL-10, M-CSF, and TGF-β. Among these, IL-4 and IL-13 primarily promote M2 polarization by activating the NF-κB/JAK/STAT6 signaling pathway [26]. This subtype highly expresses markers (CD206, CD163, Arg-1) and secretes anti-inflammatory factors (IL-10, TGF-β) and chemokines (e.g., CCL17), thereby exerting anti-inflammatory, tissue repair, and immunomodulatory functions [16]. In the endometrium, M2 macrophage numbers increase during the proliferative phase to participate in tissue regeneration and angiogenesis [27,28]. During the secretory phase and pregnancy, the CD163^+^CD206^+^ population further expands, contributing to maintaining an immune-tolerant microenvironment that supports embryo implantation and suppresses maternal–fetal rejection [29].

#### 2.2.2. Non-Classical Subtype Classification

The M1/M2 classification has evolved into a more refined paradigm, with M2 macrophages further subdivided: IL-4/IL-13-induced M2a participate in tissue repair; immune complexes combined with TLR/IL-1R agonists induce M2b (exerting immunoregulatory functions); IL-10/TGF-β-induced M2c exhibit anti-inflammatory and anti-fibrotic roles during decidualization; IL-6 combined with adenosine receptor or TLR ligands induces M2d, which promotes vascular remodeling and placental angiogenesis via VEGF [17]. This finer subcategorization of M2 subtypes deepens understanding of the functional diversity of endometrial macrophages during menstruation and pregnancy, expanding the original M1/M2 classification framework.

Notably, during pregnancy, there is a special type of decidual macrophage in the decidua, primarily located at the maternal–fetal interface, which plays roles in immune regulation, tissue remodeling, angiogenesis, and apoptotic cell clearance [30]. Current research shows that decidual macrophage function and phenotype undergo dynamic changes with pregnancy progression. During the implantation phase, M1-type decidual macrophage numbers increase significantly, facilitating embryo and placental attachment. Following trophoblast cell invasion into the uterine stroma, decidual macrophages exhibit a mixed M1/M2 phenotype—this unique physiological state supports uterine vascular remodeling to ensure adequate fetal blood supply and prevents embryo immune rejection. As pregnancy advances to the mid-to-late stages, these mixed-phenotype decidual macrophages gradually shift toward an M2-dominant profile, ultimately establishing an anti-inflammatory environment conducive to fetal development [17,18]. Further delineating this heterogeneity, research by Houser et al. identified two distinct CD14^+^ decidual macrophage subpopulations in early pregnancy: CD11c^high^ and CD11c^low^. Despite transcriptional differences, these subsets cooperate to maintain maternal–fetal immune tolerance [31], underscoring the functional complexity and heterogeneity of this cellular compartment.

Although the traditional M1/M2 macrophage classification paradigm has been extensively studied in the physiology and pathology of various tissues and organs, recent advances in single-cell sequencing and multi-omics technologies have revealed its increasing inadequacy for further research on endometrial macrophages. This binary framework is thus being replaced by novel, non-canonical classification frameworks that more precisely capture the spatiotemporal heterogeneity of macrophages and their functional dynamics in the female reproductive system [32,33].

Firstly, a metabolic reprogramming-based classification for endometrial macrophages has been proposed, defined by their distinct metabolic characteristics in response to microenvironmental signals [32]. The glycolysis-dependent (M1-like) subtype highly expresses HIF-1α, promotes pro-inflammatory cytokine secretion via aerobic glycolysis, and participates in menstrual inflammatory responses [33].The oxidative phosphorylation-dependent (M2-like) subtype primarily relies on mitochondrial metabolism, facilitating anti-inflammatory factor secretion through the PPARγ/STAT6 pathway to support pregnancy immune tolerance [34]. The fatty acid oxidation-dependent subtype exhibits high CD36 expression, promotes immunosuppressive microenvironment formation via β-oxidation, and is commonly observed in obesity-related endometrial pathologies [35].

Furthermore, single-cell RNA sequencing has revealed more refined endometrial macrophage subpopulations. Ren et al. discovered MKI67^+^ proliferative macrophages are predominantly located in the endometrial basal layer—these cells contribute to endometrial homeostasis and possess tissue-specific self-renewal capabilities [36]. Tolerogenic macrophages, characterized by LILRB4 and PD-L1 expression, play a critical role in suppressing T-cell responses and early pregnancy decidualization [37]. In contrast, migratory macrophages (CCR7^+^S100A8^+^) are closely associated with the formation of endometriotic lesions [38]. Additionally, disease-specific macrophage classification criteria provide new perspectives for characterizing endometrial physiological and pathological features [38]. Future research will focus on integrating multi-omics data to decipher dynamic transitions between macrophage subsets and explore their potential in personalized medicine. These advances are expected to promote precision diagnosis and treatment of endometrial-related diseases, bringing new breakthroughs to reproductive health.

### 2.3. Bidirectional Crosstalk Between Macrophages and Oxidative Stress

Redox homeostasis acts as a core regulator governing the phenotype and function of macrophages within the endometrial microenvironment. Physiological concentrations of reactive oxygen species (ROS) mediate cellular signal transduction, while excessive and sustained ROS production triggers oxidative stress, forming a bidirectional regulatory loop between ROS and macrophages. ROS derived from mitochondrial dysfunction, hypoxia-reoxygenation, as well as heme and iron overload can remodel metabolic programming and redirect polarization the status of macrophages. In turn, activated macrophages generate ROS and reactive nitrogen species via NADPH oxidases, mitochondrial metabolism and inducible nitric oxide synthase and secrete inflammatory mediators including TNF-α and IL-6, which further exacerbate local oxidative injury [39,40].

This bidirectional crosstalk is particularly critical in the context of female reproductive disorders. In patients with recurrent spontaneous abortion (RSA), JPT2-deficient trophoblast cells suppress the JNK/IL-6 signaling axis and remodel citrate metabolism, thereby driving macrophages toward M1 polarization and massive intracellular ROS accumulation [41]. For patients with endometriosis (EMS), repeated intraperitoneal hemorrhage leads to the buildup of heme and iron. Peritoneal macrophages isolated from these patients exhibit markedly elevated iron storage, which correlates with iron overload in peritoneal fluid, creating a self-amplifying vicious cycle consisting of oxidative stress, macrophage dysfunction and persistent chronic inflammation [42,43]. Nevertheless, longitudinal studies profiling redox dynamics across distinct human endometrial macrophage subsets remain scarce at present.

## 3. The Role of Macrophages in Cyclical Endometrial Remodeling and Pregnancy

**Figure 2:**
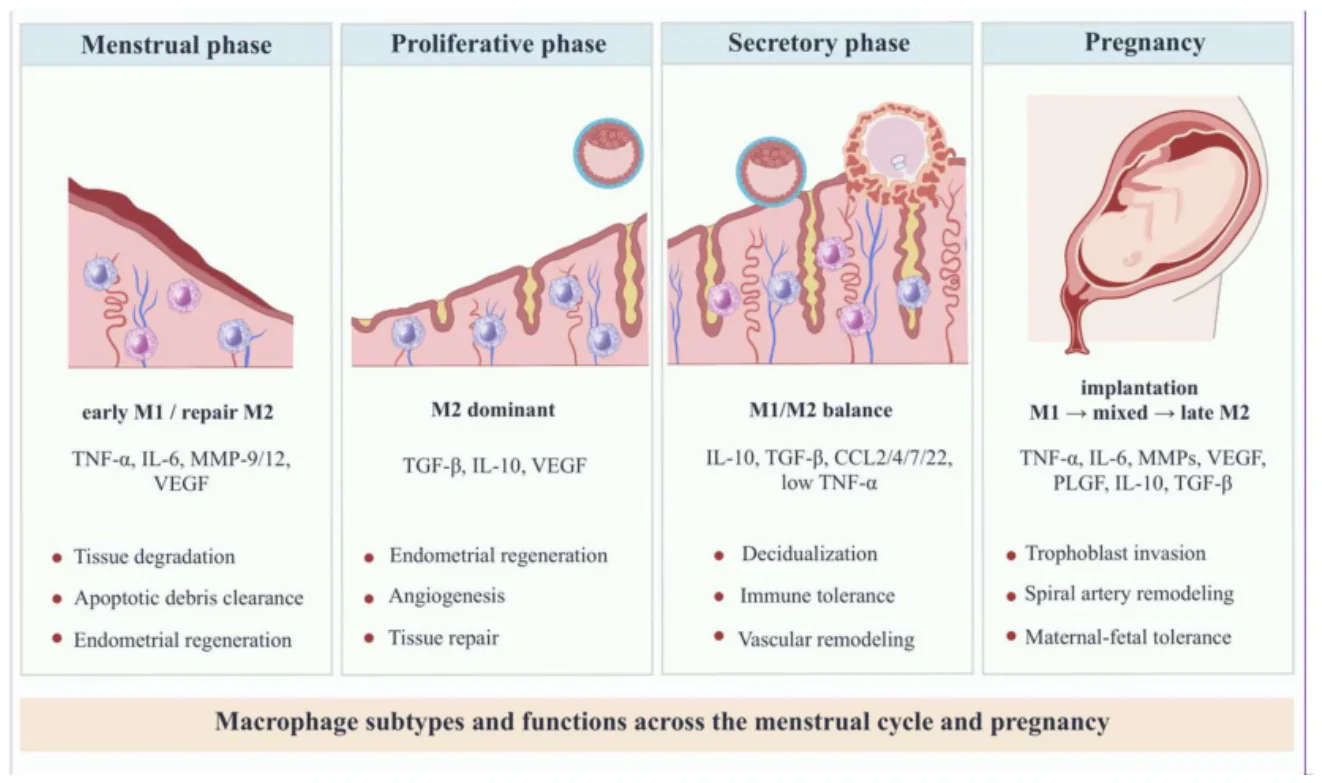
Phenotypic switch and functional profiles of endometrial macrophages across the menstrual cycle and pregnancy. Schematic illustration depicting the dynamic shifts in endometrial macrophage polarization status, key secreted mediators, and corresponding physiological functions during menstruation, proliferation, secretion, and post-implantation pregnancy, including tissue repair, decidualization, vascular remodeling, trophoblast invasion, and maternal–fetal immune tolerance. Abbreviations: TNF-α = tumor necrosis factor-alpha; VEGF = vascular endothelial growth factor; TGF-β = transforming growth factor-β; MMP = matrix metalloproteinase; PLGF = placental growth factor.

Accumulating evidence has verified that endometrial macrophages undergo stage-specific dynamic remodeling across the proliferative, secretory, and menstrual phases and gestational periods, with distinct subpopulations exhibiting unique surface marker profiles and division of labor in maintaining endometrial homeostasis. As schematically depicted in Figure 2, macrophage polarization status, secreted mediators and physiological roles shift dynamically alongside endometrial cyclic changes and embryo implantation. To systematically sort out the phenotypic classification, characteristic biomarkers and stage-dominated biological functions of macrophage subsets involved in menstrual cycle regulation and pregnancy establishment, we summarize the core information in Table 1.

### 3.1. Proliferative Phase Macrophages Involved in Regeneration

In the non-pregnant state, the endometrium undergoes regular histological changes throughout the menstrual cycle, sequentially experiencing the proliferative phase, secretory phase (including decidualization), and menstrual phase (characterized by shedding and regeneration) [53]. This process is primarily precisely regulated by ovarian-derived estradiol and progesterone [11]. During the proliferative phase, estrogen promotes endometrial regeneration via a dual mechanism: activating angiogenesis and regulating macrophage-mediated tissue remodeling. Following ovulation, progesterone becomes the dominant hormone, preparing the endometrium for potential embryo implantation by inducing tissue reorganization and stromal cell decidualization [54]. Notably, inflammatory cell populations (monocytes, macrophages, neutrophils) play a central regulatory role in this cyclic process of tissue breakdown and repair. Current studies show that macrophages account for approximately 1–2% of all endometrial cells during the proliferative phase, with their proportion dynamically fluctuating across different menstrual cycle stages [55]. It has been demonstrated that endometrial macrophages express stage-specific markers corresponding to different menstrual cycle phases. For instance, during the proliferative phase, macrophages express adhesion and activation markers (CD71, CD69, CD54), suggesting a potential role in endometrial functional layer regeneration and proliferation [56].

### 3.2. Secretory Phase Macrophages Maintain Endometrial Homeostasis

Decidualization is a crucial process in the secretory phase endometrium, induced by progesterone. It is characterized by the terminal differentiation of stromal cells into an epithelioid morphology, accompanied by secretion of various factors to modulate the microenvironment and support embryo implantation [57,58]. In non-conception cycles, decidualized tissue cannot be maintained and is ultimately shed during menstruation.

The endometrial immune microenvironment exhibits significant cyclic dynamics, with various immune cells playing crucial roles in tissue repair [59]. Studies show the proliferative phase endometrium contains only a limited number of specific immune cell subsets, including phenotypically specialized macrophages, mast cells, T lymphocytes, and uterine Natural Killer (uNK) cells [60,61]. In contrast, the secretory and menstrual phases are characterized by substantial leukocyte infiltration, supporting the hypothesis that “menstruation represents an inflammatory event” [62,63]. The proportion of endometrial macrophages fluctuates throughout the cycle: they constitute approximately 3–5% of cells in the secretory phase, increasing to 6–15% during menstruation [59]. This dynamic variation is closely associated with cyclic hormonal fluctuations. In the late secretory phase, macrophages and uNK cells can express hCG receptors [64,65], a mechanism potentially contributing to the increased numbers of these cells. Further research demonstrates that endometrial macrophages express estrogen receptor-β (ER-β) and estrogen-related receptor-β (ERR-β), suggesting their function may be regulated via estrogen-dependent pathways [48]. However, as this cell population does not express the progesterone receptor (PR), progesterone’s effects are likely mediated indirectly through other factors [66].

Studies show that the increased macrophage population during the secretory phase is closely associated with endometrial upregulation of C-C motif chemokine ligand 4 (CCL4), CCL7, CCL21, and CCL22—likely driving their cyclic recruitment [67]. Spatially, macrophages form large granular aggregates are primarily distributed in periglandular areas and accumulate in perivascular regions near the luminal epithelium prior to ovulation [53], suggesting their role in rapidly responding to tissue breakdown and coordinating repair processes. It remains unclear whether this population represents in situ retained cells or undergoes cyclic replacement. To address this question, studies have systematically characterized endometrial macrophage phenotypes using multi-marker strategies. Notably, the CD68^+^CD163^+^ macrophage subpopulation demonstrates the capacity to secrete both pro-inflammatory and anti-inflammatory cytokines, highlighting their remarkable functional heterogeneity [47].

Endometrial macrophages work in concert with T cells and uNK cells to orchestrate local inflammation and tissue remodeling, thereby maintaining endometrial homeostasis [68]. In the late secretory phase, regulatory T (Treg) cells promote M2 macrophage polarization via IL-10 and TGF-β signaling [69]. Concurrently, uNK cells drive M1 polarization through IFN-γ, enhancing their clearance capacity [23]. This coordinated interaction network balances M1 and M2 functions, preventing chronic inflammation and ensuring successful post-menstrual repair.

### 3.3. Macrophages Participate in Clearance and Remodeling During Menstruation

Menstruation refers to the cyclical shedding of the endometrial functional layer and its subsequent regeneration in the next cycle, while the basalis layer remains intact throughout [44]. In the absence of pregnancy, corpus luteum regression causes a sharp decline in progesterone levels, triggering menstruation [54]. The menstrual process involves the coordinated action of multiple systems: (1) activation of local inflammatory response; (2) upregulation of extracellular matrix (ECM)-degrading enzyme systems; (3) ischemic injury induced by spiral artery vasospasm; (4) rhythmic myometrial contractions [70]. Inflammatory cells (including macrophages) act as key mediators of both tissue breakdown and repair, accounting for up to 15% of stromal cells during menstruation and primarily participating in tissue clearance and remodeling [71].

Endometrial repair is initiated when stromal cells sense progesterone withdrawal and activate inflammatory signaling pathways, upregulating local inflammatory mediators (e.g., IL-6, TNF) and chemokines that recruit monocytes, macrophages, and neutrophils [45,72]. Beyond hormonal regulation, microenvironmental changes (e.g., hypoxia from tissue breakdown) further modulate macrophage function. Both clinical and animal studies show that hypoxic conditions promote macrophage expression of repair factors like VEGF [46]. The menstrual endometrium is in a hypoxic state, and delayed repair is associated with hypoxia—underscoring macrophages’ important role in this process [73]. Although HIF-1 inhibition in murine models did not alter macrophage numbers, whether they undergo phenotypic adaptation similar to that in the tumor microenvironment remains to be verified [73]. Macrophages facilitate ECM degradation and tissue shedding by secreting proteases (MMP-9 [74], MMP-12 [75]), while the CD163^+^CD206^+^ subpopulation is likely involved in the subsequent repair phase [29]. Neutrophils also accumulate in shedding areas, cooperating to mediate tissue breakdown. During the repair phase, macrophages localize to periglandular areas, promoting reconstruction by clearing apoptotic cells and secreting regenerative factors; bone marrow depletion experiments confirm that their absence delays repair [76,77]. CSF1R-GFP mouse model studies further demonstrate that monocyte-derived macrophages accumulate in actively repairing areas, while tissue-resident macrophages distribute in already repaired regions—indicating their cooperative regulation of regeneration [28]. These findings highlight macrophages’ central role in orchestrating the menstrual microenvironment.

### 3.4. Macrophages Participate in Stage-Specific Adaptive Immune Regulation and Tissue Remodeling During Pregnancy

A successful pregnancy relies on a delicate balance between maternal immune tolerance to the semi-allogeneic fetus and maintenance of robust anti-pathogen defense [78]—a process conceptualized as an “immune clock” [79]. The maternal immune system achieves this balance via coordinated efforts of its two main branches: innate immunity (providing rapid response and repair) and adaptive immunity (enabling antigen-specific recognition and memory) [67]. Innate immune cells (e.g., monocytes, macrophages) use pattern recognition receptors (PRRs) to identify pathogens, activate immune signaling, and eliminate threats [67]. While current research has primarily focused on the immunoregulatory roles of NK cells and T cells during pregnancy [49,50,80,81], the dynamic changes and functional regulatory mechanisms of macrophages at the maternal–fetal interface remain to be fully elucidated.

Circulating monocytes, guided by microenvironmental signals, are recruited via chemotaxis into tissues and differentiate into macrophages [82]. At the maternal–fetal interface, macrophages account for approximately 20–30% of decidual immune cells, representing the second largest leukocyte population after NK cells [83]. They interact with NK cells to jointly maintain immune microenvironment homeostasis: macrophages secrete IL-15 to activate NK cells, which in turn modulate immune responses, promote Treg cell differentiation, suppress excessive maternal T cell activation, and maintain fetal immune tolerance [51]. Furthermore, during pregnancy, macrophages are enriched in the decidua, participating in tissue remodeling and efficiently clearing apoptotic cells to prevent aberrant immune responses against fetal antigens [84].

Decidual macrophages exhibit remarkable phenotypic plasticity, playing critical roles in regulating trophoblast invasion, vascular remodeling, and maternal–fetal immune tolerance [85]. Current research has transcended the traditional M1/M2 dichotomy, revealing dynamic temporal phenotypic evolution throughout gestation: M1-dominant during implantation; transitioning to mixed M1/M2 during trophoblast invasion; and finally polarizing to M2-dominated after placental circulation establishment, thereby suppressing maternal immune rejection against the fetus [11].

Decidual macrophages participate in critical pregnancy processes, including tissue remodeling, angiogenesis, and placental expansion [52]. They promote early spiral artery remodeling by secreting VEGF and PlGF; VEGF expression is regulated by PSGs [86]. Macrophages expressing MMP-9 exhibit enhanced phagocytic activity and infiltrate perivascular areas [86]. Macrophages expressing MMP-9 exhibit enhanced phagocytic activity and are observed to infiltrate perivascular areas [86]. During parturition, uterine tissues show significant leukocyte infiltration, accompanied by markedly elevated levels of PGF2α, oxytocin receptors, and pro-inflammatory cytokines (IL-6, IL-8) [87,88]. Decidual macrophages are activated by these inflammatory signals [89], contributing to tissue remodeling and apoptotic cell clearance, thereby actively promoting labor [90,91].

## 4. Role of Macrophages in Female Reproductive System Diseases

**Figure 3:**
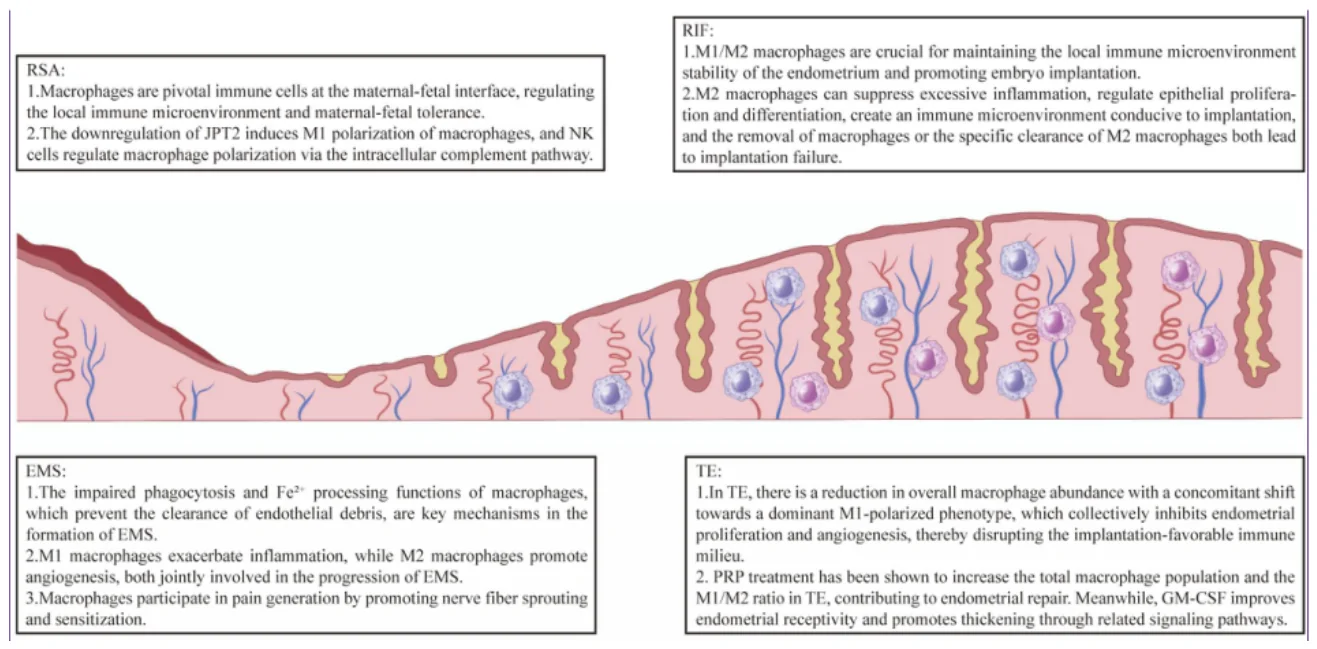
The role of macrophages in common female reproductive system disorders. Abbreviations: RSA = recurrent spontaneous abortion; RIF = recurrent implantation failure; EMS = endometriosis; TE = thin endometrium; JPT2 = Jupiter microtubule-associated homolog 2; PRP = platelet-rich plasma; GM-CSF = granulocyte-macrophage colony-stimulating factor.

As key immune cells in the female reproductive system, macrophages are widely distributed in the uterus, ovaries, fallopian tubes, and other tissues [92]. They have both immune defense and tissue repair functions: eliminating pathogens and abnormal cells to maintain microenvironment stability, and regulating inflammatory responses by secreting cytokines [93]. The imbalance of their functions is closely associated with abnormalities in the female reproductive system. This review focuses on the role of macrophages in several common female reproductive system diseases (Figure 3). To systematically sort out the phenotypic shifts and functional defects of macrophages under pathological conditions, we further summarize the aberrant polarization patterns, dysregulated surface markers and corresponding pathogenic mechanisms in recurrent spontaneous abortion, recurrent implantation failure, endometriosis and thin endometrium in Table 2.

### 4.1. RSA and Macrophages

RSA is defined as two or more consecutive pregnancy losses with the same partner, affecting approximately 5% of pregnancies and representing a major obstacle to successful gestation [104]. Currently identified etiologies for RSA include infectious factors [105], chromosomal abnormalities [106], endocrine and metabolic dysfunction [107], antiphospholipid syndrome [108], and uterine anatomical anomalies [109]. However, the cause remains unidentified in approximately 50% of RSA patients, who are classified as having unexplained recurrent pregnancy loss [110]. The underlying mechanisms of RSA are not fully elucidated, and in-depth research into its causes, regulatory molecules, and mechanisms is of great significance for disease prevention and treatment.

Macrophages account for approximately 20% of all leukocytes in the human decidua, representing the second largest leukocyte population at the maternal–fetal interface and playing a pivotal role in establishing and maintaining the local immune microenvironment [111]. Their function and phenotype are precisely regulated by the maternal–fetal interface microenvironment [22]. The maternal–fetal interface is a critical structure connecting the mother and fetus, performing essential functions such as metabolic exchange, nutrient supply, and immune defense [94]. Its integrity relies on the normal physiological activity of human extravillous trophoblast cells [95]. A successful pregnancy requires the maternal immune system to be remodeled into a state of tolerance to avoid rejection of the semi-allogeneic fetus [112]. Consequently, the various immune cells enriched at the placental-uterine junction hold a central position in pregnancy regulation [113]. Immune tolerance is maintained through an intricately coordinated cellular communication network between trophoblast cells, immune cells, and decidual stromal cells [114]. Notably, functional impairment of any of these cell types or disruptions in intercellular communication are closely associated with pregnancy disorders like RSA, although the specific molecular mechanisms still require further clarification.

A recent study demonstrated that Jupiter microtubule-associated homolog 2 (JPT2) expression is significantly downregulated in both the villous tissues of RSA patients and placentas of a mouse miscarriage model [41]. Their RNA sequencing analysis revealed that JPT2 deficiency suppresses the JNK/ACKR3 signaling axis, impairing trophoblast cell adhesion, migration, and invasion. Concurrently, it inhibits the JNK/IL-6 pathway, inducing M1 macrophage polarization and ROS accumulation. Metabolomic analysis further indicated that accumulated citrate can promote ROS generation and M1 polarization. M1 macrophages, in turn, can adversely affect trophoblast function. This study unveils a novel mechanism whereby JPT2 maintains pregnancy by regulating the immunometabolic microenvironment at the maternal–fetal interface, suggesting its potential as a therapeutic target for RSA.

Another study [115] found that decidual NK cells in RSA patients can regulate macrophage polarization via receptor-ligand interactions, and the intracellular complement system (particularly C3) plays a key role in maintaining pregnancy immune homeostasis. Specifically, ICAM1^+^ macrophages promote C3 expression in LFA1^+^ decidual NK cells. NK cell-derived cathepsin W cleaves C3 to generate C3b, which binds to VSIG4 on the macrophage surface and effectively suppresses their pro-inflammatory phenotype polarization. This research reveals a novel mechanism by which decidual NK cells regulate macrophage polarization through the intracellular complement pathway, providing a new perspective for understanding the immunopathogenesis of RSA.

### 4.2. Recurrent Implantation Failure and Macrophages

Recurrent implantation failure (RIF) is typically defined as a failure to achieve clinical pregnancy after transfer of at least three high-quality embryos or two donor oocyte-derived embryos [116]. Some cases of RIF share common etiological mechanisms with recurrent pregnancy loss (RPL) [116]. Risk factors include maternal factors (e.g., advanced maternal age, obesity), reproductive tract abnormalities (e.g., endometritis, polyps, fibroids), immune dysregulation, embryo quality, impaired endometrial receptivity, vitamin D deficiency, and genetic polymorphisms (e.g., in HLA-G, VEGF) [116,117,118,119].Furthermore, non-coding RNAs (e.g., miRNAs, lncRNAs) are implicated in RIF pathogenesis by regulating gene expression [120,121].

Vitamin D acts as an upstream regulatory molecule that links systemic nutritional status to the endometrial immune microenvironment. Components related to vitamin D metabolism and signaling pathways are expressed in reproductive tissues and immune cells such as macrophages, including the endometrium and decidua [96]. Activated macrophages express the CYP27B1 enzyme, which locally converts 25-hydroxyvitamin D_3_ into its active metabolite 1,25-dihydroxyvitamin D_3_, suggesting a bidirectional regulatory relationship between macrophage activation and vitamin D bioavailability [97]. In women with RIF, vitamin D insufficiency or deficiency is accompanied by an elevated proportion of CD68^+^ macrophages in the mid-luteal endometrium, and this immune dysregulation can be alleviated by vitamin D supplementation [122]. In vitro experiments on monocytes and macrophages of non-endometrial origin further confirm that a vitamin D dose dependently suppresses the LPS-triggered activation of p38 mitogen-activated protein kinase and reduces the secretion of IL-6 and TNF-α [123]. Other studies have demonstrated that 1,25-dihydroxyvitamin D_3_ facilitates M2 polarization in experimental macrophage models [124]. Nevertheless, current evidence fails to define a linear dose–response relationship for the regulation of human endometrial macrophage aggregation and polarization, nor has the optimal supplementation dose been determined.

Successful embryo implantation relies on the precise interaction between the embryo and the endometrium. For RIF patients with high-quality embryos, the local immune microenvironment status of the endometrium may be a key factor influencing implantation outcome [125]. During the window of implantation, the endometrium, under hormonal regulation, secretes various cytokines and chemokines that recruit and activate immune cell populations (uNK cells, macrophages, dendritic cells, T cells, B cells) [126]. These cells cooperate to regulate the balance between immune tolerance and inflammatory responses, thereby promoting embryo implantation and pregnancy maintenance [84]. However, the specific roles and molecular mechanisms of these diverse immune cells in the implantation process remain debated and require further in-depth investigation.

Macrophages are mainly divided into pro-inflammatory M1 type and anti-inflammatory M2 type [127]. Studies show that in CD11b-diphtheria toxin receptor mice, clearing CD11b+cells (including M1 and M2 macrophages) with low-dose diphtheria toxin causes embryo implantation failure [98]. Further mechanistic exploration revealed that this implantation defect is related to luteal dysfunction and decreased peripheral blood progesterone levels [98]. In the CD206-diphtheria toxin receptor model, specific clearance of M2 macrophages still causes implantation defects, despite normal corpus luteum morphology and serum progesterone levels [128]. This result indirectly suggests that M1 macrophages may play an important role in maintaining normal luteal function and progesterone levels. To investigate the cause of implantation failure due to M2 deficiency, researchers transplanted embryos from M2-depleted female mice into pseudopregnant recipients and found that embryos still implanted normally, indicating embryo quality was not the main cause [128]. Further analysis showed increased Ki67 (proliferation marker) expression in uterine epithelial cells of M2-deficient mice, as well as significantly elevated Wnt4, Wnt7B, and β-catenin expression, suggesting the Wnt/β-catenin pathway may mediate epithelial hyperproliferation [128]. Additionally, after clearing M2 macrophages, the local uterine microenvironment favors M1 polarization, accompanied by increased TNF-α mRNA expression, indicating enhanced inflammatory response [128]. In summary, M2 macrophages may promote successful embryo implantation by suppressing M1-mediated excessive inflammation and precisely regulating epithelial proliferation and differentiation to create an immune microenvironment suitable for embryo implantation [99].

### 4.3. EMS and Macrophages

EMS is a common estrogen-dependent chronic inflammatory condition, affecting approximately 10–15% of reproductive-aged women worldwide, with hallmark features including pelvic pain and infertility [129]. EMS pathogenesis is closely associated with the “retrograde menstruation” theory: endometrial fragments reflux into the peritoneal cavity, then adhere, implant, and grow to form ectopic lesions [130,131]. Macrophages play a complex and central role in EMS initiation and progression, leading some researchers to label it a “macrophage-mediated disease” [132]. Studies consistently show significant alterations in the number and function of macrophages in the peritoneal fluid and ectopic lesions of affected individuals [133,134].

In patients with EMS, the diminished expression of scavenger receptors (e.g., CD36, CD163) on macrophages impairs their phagocytic function, preventing the timely clearance of refluxed endometrial fragments and thereby enabling their survival and the establishment of ectopic lesions [135]. Furthermore, the capacity of macrophages to process iron (Fe^2+^) derived from erythrocytes is dysregulated [100]. Excessive Fe^2+^ accumulation can induce cytotoxicity, leading to macrophage apoptosis and further compromising their clearance capacity [136]. Studies have shown that the peritoneal fluid of EMS patients contains a large number of iron-overloaded macrophages, which also exhibit reduced adhesion and migratory function, creating conditions conducive to the ectopic attachment of endometrial fragments [137,138].

The polarization state of macrophages is crucial in endometrial-related pathologies. Under normal conditions, M2 macrophages predominate in the endometrium, maintaining an anti-inflammatory microenvironment and supporting embryo implantation [139]. In contrast, EMS is characterized by a shift in macrophage polarization towards a pro-inflammatory phenotype, secreting inflammatory factors such as IL-1, IL-6, IL-8, and MCP-1, which exacerbate inflammation and promote the growth of ectopic lesions [140,141]. Furthermore, within the peritoneal cavity and ectopic lesions, M2-polarized macrophages contribute to disease progression by secreting factors like VEGF-A and TGF-β to promote angiogenesis and tissue repair, and by enhancing cell survival and proliferation via the STAT3 signaling pathway [142,143].

Pain is one of the primary clinical manifestations of EMS, and macrophages contribute to its pathogenesis through multiple mechanisms. They facilitate abnormal nerve fiber sprouting and sensitization by forming close contacts with nerve fibers within the lesions and secreting factors such as Netrin-1 and IGF-1, thereby amplifying pain signaling [101,102,144]. Clinical studies have shown that the ratio of CD14high to CD14low macrophage subsets in the peritoneal fluid correlates significantly with pain scores [103].

Furthermore, exosomes derived from macrophages and the long non-coding RNAs they carry (e.g., CHL1-AS1) can regulate gene expression, promoting the proliferation, migration, and invasion of ectopic endometrial cells [145]. Certain cytokines, such as IL-33, can enhance the survival of ectopic cells by inhibiting ferroptosis through corresponding signaling pathways [146].

In summary, macrophages play a dual role in EMS: they are involved in clearing refluxed endometrial debris, yet their functional impairment and aberrant polarization also contribute to lesion establishment, vascularization, innervation, and pain generation. In-depth investigation of macrophages and their associated signaling pathways holds promise for identifying novel targets for the diagnosis and treatment of this disease.

### 4.4. Thin Endometrium and Macrophages

Thin endometrium (TE), characterized by an endometrial thickness of ≤7 mm, represents a pathological condition and a significant cause of embryo implantation failure and infertility [147,148]. Various factors contribute to its pathogenesis, including intrauterine procedures, chronic inflammation, inadequate estrogen response, and insufficient uterine blood perfusion [148].

In recent years, cellular and molecular dysregulation has been recognized as a key mechanism in the pathophysiology of TE. Growing evidence indicates a strong link between TE and functional abnormalities of macrophages. As central regulators of the endometrial immune microenvironment, macrophages play crucial roles in maintaining endometrial homeostasis, tissue repair, and angiogenesis. In the context of TE, factors like chronic inflammation can disrupt macrophage function, typically manifesting as enhanced M1 polarization. This shift subsequently inhibits endometrial cell proliferation and angiogenesis, while simultaneously disrupting the immune-tolerant microenvironment essential for successful embryo implantation.

Lv et al., utilizing single-cell RNA sequencing, demonstrated a significant reduction in macrophage numbers within thin endometrium (TE) [149]. This depletion impairs the supportive role for stromal cell growth, thereby exacerbating inadequate endometrial proliferation. In vitro experiments further confirmed that macrophage-conditioned medium significantly promotes the proliferation of human endometrial stromal cells (hESCs), indicating that macrophages are a key immune population regulating endometrial regeneration and repair [150]. Research by Zeng et al. showed that autologous platelet-rich plasma (PRP) treatment increases the total macrophage population in TE and significantly elevates the proportion of the M1 subtype. M1 macrophages were found to be enriched in pathways related to innate immunity and inflammation, potentially contributing to endometrial repair by secreting cytokines that recruit stem cells and promote angiogenesis. Immunohistochemical results corroborated this, showing increased expression of the M1 marker iNOS following PRP treatment [151]. Furthermore, granulocyte-macrophage colony-stimulating factor (GM-CSF) has shown therapeutic potential for TE. Mao et al. reported that intrauterine perfusion of GM-CSF significantly increased endometrial thickness and improved clinical pregnancy rates in patients [152]. Wei et al., using a rat TE model, confirmed that GM-CSF upregulates HOXA10 expression by activating the MAPK/ERK signaling pathway, thereby improving receptivity and promoting endometrial thickening; knocking down HOXA10 abolished this therapeutic effect. Their study also indicated that GM-CSF enhances cell adhesion, promotes angiogenesis, and inhibits apoptosis [153].

In summary, the association between TE and macrophages is primarily reflected in the regulation of the immune microenvironment, inflammatory responses, tissue repair, and angiogenesis. Although current research in TE remains largely focused on areas such as stem cell transplantation, growth factors, and hormonal therapies, the role of macrophages as core participants in immune regulation and tissue repair during the development and progression of TE requires further in-depth exploration. Targeting macrophages for immunomodulation has thus emerged as a promising novel direction in TE therapy [154,155].

## 5. Conclusions

Macrophages are central regulators of cyclical endometrial remodeling, menstrual repair, implantation, pregnancy maintenance, and reproductive immune homeostasis. Single-cell sequencing and multi-omics have revealed heterogeneity beyond the conventional M1/M2 framework, while spatial analyses define tissue niches. Animal lineage-tracing and perturbation models provide mechanistic evidence, whereas in vitro systems and human specimens enhance clinical relevance.

However, each approach has limitations. Most human studies are cross-sectional, involve small, heterogeneous cohorts, and are affected by reproductive stage, sampling location, treatment history, and tissue processing. Single-cell studies may lose spatial information and often infer function from transcriptional signatures without direct validation. Animal models do not fully reproduce human menstruation or pregnancy, and macrophage-depletion markers are not always subset-specific. Moreover, contradictory findings remain unresolved: M1-like programs are associated with pregnancy loss, implantation failure, and EMS, but may contribute to endometrial repair after platelet-rich plasma treatment; conversely, M2-like macrophages support regeneration and maternal–fetal tolerance but may promote vascularization, lesion persistence, and pain in endometriosis. These observations indicate that macrophage effects depend on lineage, location, activation intensity, disease stage, and timing. The principal conceptual bottleneck is the use of static M1/M2 labels and limited markers as proxies for function. The origins, transition trajectories, causal roles, and reproducible signatures of endometrial macrophage subsets remain insufficiently defined.

Future studies should prioritize standardized multicenter longitudinal sampling across reproductive stages and integrate single-cell, spatial, proteomic, epigenomic, and metabolic data. Candidate macrophage states should be validated using lineage-resolved models, patient-derived organoids or assembloids, ex vivo tissues, and selective perturbation and linking them to implantation, pregnancy loss, endometrial regeneration, pain, and treatment response. In our view, progress in macrophage biology could shift diagnostics from cell counts or M1/M2 ratios toward stage-specific signatures incorporating cellular state, spatial distribution, and secreted mediators. Therapy should likewise move from global depletion or indiscriminate polarization toward biomarker-guided, temporally controlled, and locally delivered modulation, with an assessment of antimicrobial defense, tissue repair, and maternal–fetal safety. More broadly, the endometrium offers a model for understanding tissue-resident immunity, immune tolerance, and inflammation–resolution transitions, connecting advances in reproductive medicine with fundamental immunology.

## Figures and Tables

**Table 1:** Subpopulations, phenotypic markers, functional characteristics and corresponding references of endometrial macrophages regulating menstrual cycle and pregnancy.

Physiological Stage	Macrophage Subsets	Core Phenotypic Markers	Main Biological Functions	References
Menstrual phase	Predominantly classically activated M1 macrophages, with a small number of repair-oriented M2 macrophages	M1: CD80, CD86, iNOS, TLR4 M2: CD163, CD206, Arg-1	1. M1: Secrete TNF-α, IL-6, MMP-9/12 to degrade extracellular matrix, mediate endometrial shedding and eliminate apoptotic tissue debris 2. M2: Produce VEGF and TGF-β to trigger endometrial repair and angiogenesis 3. Adapt to hypoxic microenvironment and sustain tissue remodeling homeostasis	[44,45,46]
Proliferative phase	TRMs + bone marrow-derived M2 macrophages	CD68^+^, CD163^+^, CD206^+^, CD71, CD69, CD54	1. Secrete pro-angiogenic factors to promote endometrial gland and stromal proliferation 2. Clear residual apoptotic cells after menstruation and initiate endometrial repair 3. Release TGF-β and IL-10 to maintain a mild anti-inflammatory microenvironment	[28,41,47]
Secretory phase (pre-implantation window)	Mixed M1/M2 macrophages; CD163^+^CD68^+^ double-positive subset	CD68, CD163, ER-β, receptors for CCL4/7	1. Regulate stromal cell decidualization to prepare for embryo implantation 2. Coordinate with uNK cells to balance local inflammation and build an immune-tolerant microenvironment 3. Mediate chemotactic recruitment of immune cells and remodel endometrial vasculature	[40,43,48]
Early pregnancy (implantation stage)	M1-dominant decidual macrophages	CD80, iNOS, HIF-1α	1. Moderate inflammatory response facilitates trophoblast adhesion and invasion 2. Regulate early spiral artery remodeling to establish the foundation for maternal–fetal substance exchange	[31,49,50]
Mid-to-late pregnancy	M2-dominant decidual macrophages	CD163, CD206, LILRB4, PD-L1, Arg-1	1. Secrete IL-10 and TGF-β to suppress maternal allogeneic rejection and maintain maternal–fetal immune tolerance 2. Continuously clear apoptotic trophoblast cells to support placental development 3. Release VEGF and PlGF to promote placental angiogenesis	[51,52]
Labor onset stage	M1-polarized macrophages	CD86, iNOS, TNF-α, IL-6	Secrete abundant pro-inflammatory cytokines to induce myometrial contraction and initiate labor	[32]

Abbreviations: TRMs = tissue-resident macrophages; iNOS = inducible nitric oxide synthase; TLR4 = toll-like receptor 4; Arg-1 = arginase-1; VEGF = vascular endothelial growth factor; TGF-β = transforming growth factor-β; MMP-9/MMP-12 = matrix metalloproteinase 9/12; TNF-α = tumor necrosis factor-α; IL-6 = interleukin-6; ER-β = estrogen receptor-β; CCL4/CCL7 = C-C motif chemokine ligand 4/7; uNK cells = uterine natural killer cells; LILRB4 = leukocyte immunoglobulin-like receptor B4; PD-L1 = programmed death ligand 1; PlGF = placental growth factor; HIF-1α = hypoxia-inducible factor 1α.

**Table 2:** Abnormal phenotypes and dysfunction of macrophages in four common female reproductive disorders.

Disease Category	Polarization Shift Feature	Dysregulated Markers	Manifestations of Functional Disorders	References
RSA	Excessive M1 polarization and suppressed M2 phenotype; disrupted decidual NK-macrophage regulatory axis	Upregulated: CD80, iNOS, TNF-α, IL-6Downregulated: CD163, VSIG4	1. Reduced JPT2 activates JNK/IL-6 axis to drive M1 shift and massive ROS accumulation, impairing trophoblast adhesion and invasion 2. Blocked intracellular complement C3b-VSIG4 pathway fails to restrain pro-inflammatory macrophage phenotype 3.Excessively pro-inflammatory microenvironment triggers maternal–fetal rejection and disturbs embryonic development	[94,95]
RIF	Insufficient M2 macrophage population and persistent M1-mediated chronic inflammation	Decreased M2 markers: CD206, Arg-1 Elevated M1 markers: TNF-α, Ki67, Wnt pathway-related proteins	1. M2 deficiency fails to inhibit Wnt/β-catenin pathway, leading to excessive uterine epithelial proliferation and impaired endometrial receptivity 2. Sustained M1-derived pro-inflammatory cytokines interfere with embryo adhesion and implantation; specific depletion of M2 macrophages directly causes implantation failure 3. Impaired corpus luteum function and insufficient progesterone further deteriorate the implantation microenvironment	[96,97]
EMS	Impaired macrophage clearance capacity, overall shift toward pro-inflammatory M1 phenotype; ectopic lesion-resident M2 macrophages abnormally promote fibrosis	1. Peritoneal macrophages downregulate scavenger receptors CD36 and CD163 2. Lesion macrophages highly express IL-6, MCP-1, VEGF, TGF-β	1. Defective phagocytosis prevents clearance of retrograde endometrial debris, enabling persistent lesion colonization 2. Disordered iron metabolism and iron overload induce macrophage apoptosis, further weakening tissue clearance function 3. M1-secreted inflammatory factors aggravate pelvic pain; lesion M2 macrophages facilitate angiogenesis and aberrant nerve sprouting, exacerbating dysmenorrhea and infertility	[98,99,100]
TE	Markedly reduced total macrophage count, loss of homeostatic M2 macrophages; transient M1 elevation participates in tissue repair after PRP intervention	Global reduction: CD68, CD163 Elevated after PRP treatment: iNOS (M1 marker)	1. Macrophage depletion leads to insufficient secretion of proliferative factors, inhibiting endometrial stromal cell proliferation and resulting in thin endometrium 2. Chronic inflammation drives persistent M1 polarization, suppressing angiogenesis, disrupting immune tolerance and reducing endometrial receptivity 3. GM-CSF remodels macrophage function, upregulates HOXA10 and facilitates endometrial repair and thickening	[101,102,103]

Abbreviations: RSA = recurrent spontaneous abortion; RIF = recurrent implantation failure; EMS = endometriosis; TE = thin endometrium; iNOS = inducible nitric oxide synthase; Arg-1 = arginase-1; VEGF = vascular endothelial growth factor; TGF-β = transforming growth factor-β; TNF-α = tumor necrosis factor-α; IL-6 = interleukin-6; MCP-1 = Monocyte Chemoattractant Protein-1; GM-CSF = granulocyte-macrophage colony-stimulating factor; HOXA10 = homeobox A10; VSIG4 = V-set and immunoglobulin domain containing 4; ROS = reactive oxygen species; Wnt = Wingless and INT-1; PRP = platelet-rich plasma; JPT2 = Jupiter microtubule-associated homolog 2.

## Data Availability

No new data were created or analyzed in this study. Data sharing is not applicable to this article.

## Abbreviations

The following abbreviations are used in this manuscript:

NK cells	natural killer cells
TRMs	tissue-resident macrophages
EMPs	yolk sac-derived myeloid progenitors
CMPs	common myeloid progenitors
GMPs	granulocyte-monocyte progenitors
PAMPs	pathogen-associated molecular patterns
LPS	lipopolysaccharide
uNK cells	uterine natural killer cells
ER-β	estrogen receptor-β
ERR-β	estrogen-related receptor-β
PR	progesterone receptor
CCL	C-C motif chemokine ligand
Treg cells	regulatory T cells
ECM	extracellular matrix
RSA	recurrent spontaneous abortion
JPT2	Jupiter microtubule-associated homolog 2
ROS	reactive oxygen species
RIF	recurrent implantation failure
RPL	recurrent pregnancy loss
EMS	endometriosis
TE	thin endometrium
hESCs	human endometrial stromal cells
PRP	platelet-rich plasma
GM-CSF	g ranulocyte- m acrophage c olony- s timulating f actor
VEGF	vascular endothelial growth factor
TGF-β	transforming growth factor-β
TNF-α	tumor necrosis factor-α
MMP	matrix metalloproteinase
PLGF	placental growth factor
LILRB4	leukocyte immunoglobulin-like receptor B4
PD-L1	programmed death ligand 1
MCP-1	monocyte chemoattractant protein-1
HOXA10	homeobox A10
VSIG4	V-set and immunoglobulin domain containing 4
iNOS	inducible nitric oxide synthase
TLR4	toll-like receptor 4
Arg-1	arginase-1
